# Supplementary material for: Trace amine-associated receptor gene polymorphism increases drug craving in individuals with methamphetamine dependence
Source: PLoS One. 2019 Oct 10;14(10):e0220270. doi: 10.1371/journal.pone.0220270 (PMC6786581; doi:10.1371/journal.pone.0220270)
Supplement: S1 Statistical Supplement — (DOCX) [file pone.0220270.s001.docx]

**SUPPORTING INFORMATION**

**S1 Statistical Supplement.** Calculation of effect size of *TAAR1* genotype and *post-hoc* power

Methods and Results:

We used the approach of Cohen [Cohen, J. (1988). Statistical Power Analysis for the Behavioral Sciences (2nd ed.). Hillsdale, NJ: Lawrence Erlbaum Associates, Publishers]. We ran two multiple regression models in order to calculate the effect size of *TAAR1* genotype. Model 1 (M_1_) used craving as the dependent variable with Group, Age, Education and Gender as predictors. Model 2 (M_2_) added Genotype to the set of predictors. We then calculated

$f^{2}=\frac{R_{M_{1}}^{2}-R_{M_{2}}^{2}}{1-R_{2}^{2}}$,

where $R_{M_{1}}^{2}$and $R_{M_{2}}^{2}$are squared multiple *R* for the two models and *f* is a measure of the effect size of adding genotype to the model. In this case, $R_{M_{1}}^{2}$= 0.227, $R_{M_{2}}^{2}$= 0.286 and *f* = 0.29, corresponding to Cohen’s *d* = 2*f* = 0.58, a medium effect size. We then calculate

$L=f^{2}(N-k-1)$,

Where *N* is the number of subjects and *k* is the number of model degrees of freedom. We then used the value of *L* in table E.2 to interpolate a *post hoc* power of 0.36.

Discussion:

While the sample size for this investigation is small for genetic studies, there was, nevertheless, a statistically significant moderate effect (d = 0.58) of genotype on craving demonstrated in the MA groups. As this effect size is associated with a power of about 0.4 for this number of subjects, it is not unlikely that the effect was detected. Nonetheless, confirmation of the genotype effect with larger samples would increase confidence in this finding. For follow-up, confirmatory studies the predicted number of subjects to reach a power of 0.8 with this effect size is 150.
