# Supplementary material for: Trace amine-associated receptor gene polymorphism increases drug craving in individuals with methamphetamine dependence
Source: PLoS One. 2019 Oct 10;14(10):e0220270. doi: 10.1371/journal.pone.0220270 (PMC6786581; doi:10.1371/journal.pone.0220270)
Supplement: Attachment — Submitted filename: Response to Reviewers.pdf [file pone.0220270.s002.pdf]

U.S. Department of Veterans Affairs

VA PORTLAND HEALTH CARE SYSTEM
3710 SW U.S. Veterans Hospital Rd.
Portland, OR 97239

August 28, 2019

Dear Dr. Weinstein,

The reviewers of our manuscript provided helpful comments, and we hope you agree that our responses have substantially improved our submission.

Reviewer #1:
"This is an interesting research that focuses on putative genetic variants of methamphetamine addiction, which has a high impact on individual's health and substantial burden for the society. The paper is in general well written. The methodology utilized and statistical analyses performed sounds adequate and the results provided may be of great interest for researchers in the field of the genetic determinants of drug addictions, in particular for psychostimulants, opening a door on the development of innovative pharmacological therapies having the TAAR1 receptor as a target for these pathological conditions. The reference list covers relevant and in-time research. The discussion of the results is adequate and well inserted in the contest of the current research in the field. It is appreciable that the authors recognize some of the major limits of that work (i.e., cross-sectional study, small sample). Actually, I do not have substantial criticism on this interesting and well conducted work. However, the authors can find below a short list of suggestions/questions that, in my opinion, if addressed may strengthen the paper."

We thank the reviewer for these encouraging general comments and below have responded point-by-point to the specific comments.

1. *"The title is someway misleading. In accordance with the data obtained, I suggest to change it in 'Trace amine-associated receptor gene polymorphism increases drug craving in methamphetamine dependent individuals' or something similar, accounting for the fact that the significant association with craving has been found only in meth users after addiction has developed."*

We like the suggestion of a more specific title and accepted the suggestion.

2. *"It would be better provide the reader of a short description of the neuropsychological tests used instead of redirecting them to the cited references."*

Brief descriptions of the neuropsychological tests for those not face-valid, in addition to references, have been provided in the revised manuscript (please see Procedures).

3. *"Due to the strong association between psychostimulants addictions and psychotic symptoms, I am wondering why a neuropsychological test on psychotic symptoms (or something similar) has not been included in the study?"*

We used the MINI structured interview to identify psychotic symptoms. We also note that subjects with active psychosis were excluded. This has been clarified in the Materials and Methods section.

4. *"Some explanation on the exclusion of nicotine and caffeine as addictive drugs in the inclusion/exclusion criteria should be provided. In particular, nicotine is a potent addictive drug and several genetic variants were found to be linked to its addiction and relapse."*

Nicotine and caffeine use are ubiquitous in substance-using populations. Excluding subjects who used these drugs would have eliminated all of the MA group subjects. We have added to the Discussion section to note that the frequency of smoking did not differ between V288V and wild type groups.

5. *"Some comment on the putative brain areas involved in the observed differences and putatively responsible for the higher craving in the CV group could be provided to the reader."*

We hesitate to speculate excessively on specific brain regions, as we did not measure any *in vivo* imaging in this report. We note, however, in the discussion that TAAR1 has a prominent role on dopamine projections to the striatum and within the ventral tegmental area and that these regions influence the neurocircuitry of craving.

<u>Reviewer #2</u>:

"Overall, the results of the study are interesting, and novel too - as the authors point out that this is the first study of the association between this SNP and methamphetamine craving in humans."

We thank the reviewer for their positive comments and below have responded point-by-point to the specific comments.

1. *"The first sentence starts with a bang, with an attention-grabbing statement about the growing use of meth; but the reference included may not be the most scientifically valid one. Perhaps include an additional reference (e.g.https://www.ncbi.nlm.nih.gov/pubmed/23273775) to complement the existing one."*

We agree that some peer-reviewed epidemiological references would be helpful and strengthen the paper. Accordingly, we have added two sentences and three references to the Introduction.

2. *"Also in the introduction, the authors state that 'Polymorphisms in several genes are associated with drug dependence, including genes encoding opioid receptors...' The authors should probably specify which drugs are associated with these polymorphisms."*

A detailed discussion of addiction genetics is beyond the scope of this report. We have included, in addition to the specific examples with opioid addiction, a reference to a review of addiction genetics (*i.e.*, Agrawal A, Edenberg HJ, Gelernter J. Meta-Analyses of Genome-Wide Association Data Hold New Promise for Addiction Genetics. J Stud Alcohol Drugs. 2016;77(5):676-80).

3. *"In the Methods, were the controls recruited systematically from a different location than the drug users? The authors don't seem to mention SES, which may be a potential concern."*

Controls were recruited, primarily on-line or through newspaper listings, from the same Portland metro area as the individuals with MA dependence. We excluded subjects with greater than an associate's degree to lessen differences in socioeconomic status. This information has been added to the revised manuscript.

4. *“How was history of prior medical illness determined? Was it by self-report - if so, please specify?”*

Subjects’ medical histories were determined by self-report, now noted in the revised manuscript.

5. *“Details about the urine drug screens should be provided, as they are not all the same.”*

We thank the reviewer for raising this point. Urine drug screens were performed with Uscreen 6 panel Drug Test Cups (RapidDetectINC, Poteau, OK) (please see revised Methods section). The test screens for cocaine, marijuana, opioids, amphetamine, methamphetamine and benzodiazepines and was read for qualitative results on-site. No confirmation tests were performed.

6. *“Importantly, who conducted the interviews to determine if subjects met criteria for DSM-IV diagnoses, and how were they qualified and/or trained? If multiple interviewers were used, do we know their inter-rater reliability?”*

The following has been added to ‘Procedures’: Interviews were conducted by trained research associates under the direction of neuropsychologist Marilyn Huckans, PhD. Diagnoses and interpretation of responses to structured interviews were discussed at weekly meetings with Dr. Huckans, but no formal inter-rater reliability measures were calculated.

7. *“Please provide more details about the VAS scales used to measure craving. Are these standardized scales that have been validated?”*

More information has been added about the VAS scales used, including a reference which reports on the correlations between single-item VAS and a multiple-question Likert-type scale.

8. *“Where were procedures (including interviews and venipuncture) conducted?”*

Interviews and venipuncture were conducted at the VA Portland Health Care System. This information has been added to ‘Procedures’.

9. *“A bit more detail should be provided about the nonlinear regression used to analyze CHO data.”*

A reference for the standard curve fitting software, GraphPad Prism (San Diego, CA), is provided in the revised manuscript.

10. *“In the Results, the authors refer to the variable of "race". I believe that "ethnicity" is now the more preferred term.”*

Thank you for this feedback. “Race” has been changed to “ethnicity”.

11. *“Was there a gene dose response: in other words, did homozygotes for the SNP show greater responses?”*

The reviewer raises a good question. There were only two CV homozygotes in the MA group, and this was insufficient to determine a gene dose effect.

12. *"Probably the only major concern about this study is the relatively small sample size for the groups (including as few as 11 and 13 in two groups). This is obviously small for a genetic study...the authors are still able to eke out an effect for the PC3 factor on craving. The authors need to address this in more detail. It is briefly mentioned as a limitation, but it needs additional discussion - potentially including a power analysis of some form. I think that - on balance - the rigor used to separate the groups, combined with the detailed phenotype of the subjects, has led to a study of interest. But this last limitation is important, and should be addressed further."*

The effect size (Cohen's d) for genotype in the multiple regression model was moderate (0.58). As this corresponded to a *post hoc* power of approximately 0.5, we do not think it was unlikely that we detected this effect, even though our sample size was small. We added the calculation of effect size to the Results section and commented on sample size in this context in the Discussion section. In addition, we added *post hoc* power calculations as Supplementary Information.

Thank you again for the careful review of our manuscript.

Sincerely,

Jennifer M. Loftis, Ph.D.
